# The Injured Liver Induces Hyperimmunoglobulinemia by Failing to Dispose of Antigens and Endotoxins in the Portal System

**DOI:** 10.1371/journal.pone.0122739

**Published:** 2015-03-31

**Authors:** Wen Ting Liu, Ying Ying Jing, Zhi Peng Han, Xiao Ning Li, Yan Liu, Fo Bao Lai, Rong Li, Qiu-Dong Zhao, Meng-Chao Wu, Li-Xin Wei

**Affiliations:** 1 Tumor Immunology and Gene Therapy Center, Eastern Hepatobiliary Surgery Hospital, The Second Military Medical University, Shanghai, China; 2 Department of Comprehensive Treatment, Eastern Hepatobiliary Surgery Hospital, The Second Military Medical University, Shanghai, China; 3 Health Quarantine and Supervision Department, Fujian Entry-Exit Inspectation & Quarantine Bureau of P.R.C., Fuzhou, China; Drexel University College of Medicine, UNITED STATES

## Abstract

Hyperimmunoglobulinemia is frequently observed in patients with chronic liver diseases. However, the exact mechanism underlying the high level of antibody formation is not fully understood. In our study, we provide evidence for the functional role of the liver and the stimulation of plasma cell proliferation in hyperimmunoglobulinemia. We collected sera from patients with chronic liver diseases, and the level of serum immunoglobulins in patients was examined; this was also investigated in animal models of liver cirrhosis and hepatocellular carcinoma. An end-to-side microsurgical portacaval shunt was used to mimic liver dysfunction in rats. We used portal vein serum and inferior vena cava serum to immunize healthy rats and mice in order to confirm the function of the healthy liver in disposing of antigens and endotoxins from the gut. For the analysis of the state of plasma cell activation, plasma cells from mice were stained with PE-conjugated anti-CD138 and FITC-conjugated anti-BrdU for flow cytometry analysis. Hyperimmunoglobulinemia was observed both in patients with chronic liver diseases and in related animal models, and high plasma LPS levels were also observed. There was a significant increase in the activation and proliferation of plasma cell in mice immunized with antigens or LPS-positive serum compared with controls that were immunized with antigens and LPS-negative serum. We confirmed that the healthy liver plays an important role in disposing of antigens and endotoxins derived from the gut. Hyperimmunoglobulinemia in chronic liver diseases mainly arises due to the collateral circulation secondary to portal hypertension, gut antigens and endotoxins that bypass the liver and reach the antibody-producing cells.

## Introduction

The liver is the largest organ in the body, and its blood supply consists of two parts. 80% comes from the gut through the portal vein, and the remaining 20% is from vascularization through the hepatic artery [1]. Portal venous blood contains the products of digestion, along with antigens and microbial products that originate from bacteria in the small and large intestine [1,2]. The liver relies on its own immune system to protect itself from damage due to these toxic agents. Evidence suggests that the liver acts as an immunologic organ that plays an important role in the body’s immune response [1]. Liver endothelial cells, Kupffer cells and immune cells (such as macrophages, natural killer, natural killer T, and γδ T cells) are abundant in the innate immune system of the liver [3]. In a healthy liver, the Kupffer cells are chiefly responsible for the removal of antigenic material; most antigens are ultimately taken up by the Kupffer cells and disposed of in the liver [4].

Clinically, increased antibody production is a common diagnostic feature of patients affected with portal hypertension, hepatic cirrhosis and other liver diseases [5]. Characteristic patterns of elevation in serum immunoglobulins are observed in specific liver diseases such as autoimmune hepatitis (elevated IgG), primary biliary cirrhosis (elevated IgM) and alcoholic liver disease (elevated IgA). In alcoholic liver disease, elevated serum IgA levels are associated with more advanced liver fibrosis [6–9]. In addition, sera from patients with cirrhosis contain enhanced antibody activity to E.coli and bacteria [10,11]. However, the exact mechanism underlying the high level of antibody formation is not fully understood, but two general theories have been postulated. One is that the diseased liver fails to sequester or inactivate antigens and endotoxins absorbed from the gut because they bypass the liver via the collateral circulation, and consequently antigens and endotoxins become available to antibodies [12,13]. Another theory is that in the state of generalized immunologic reactivity, the level of immunoglobulin is elevated due to the non-specific activation of many different clones of antibody producing-cells that secrete immunoglobulins [14,15]. Additionally, several studies have demonstrated that increased levels of circulating immune globulins are associated with chronic hepatitis B virus (HBV) infection [16–18].

In the present study, an evaluation of circulating immunoglobulin in both patients and animal models affected with cirrhosis and hepatocellular carcinoma (HCC) was performed. We confirmed that a quantitative difference exists in serum immunoglobulins among normal patients and patients with cirrhosis and HCC, as well as in animal models. An end-to-side microsurgical portacaval shunt produces chronic hepatic insufficiency in rats [19]. This was used to mimic liver dysfunction, and the serum immunoglobulins were tested. We used portal vein blood (untreated by the liver) and inferior vena cava blood (treated by the liver) to immunize healthy rats or mice to confirm healthy liver function in disposing of the antigens and endotoxins from the gut. The results support the hypothesis that the hepatic "filtering" of enteric antigens and endotoxins is etiologic in initiating polyclonal antibodies, and this phenomenon is related to the activation and proliferation of plasma cells.

## Materials and Methods

### Patients

Sixty-four patients with chronic liver diseases from the Eastern Hepatobiliary Surgery Hospital of the Second Military Medical University who were treated between 2008 and 2012 were included in the study. This population consisted of 47 males and 17 females. Twenty-six patients had liver cirrhosis and 38 had HCC, and these patients consisted of 22 patients with chronic HBV infection and 16 patients who were HBV-negative (Table 1). The diagnosis of cirrhosis, HCC and HBV infection was based on clinical, biochemical, immunological and histological features. There was no evidence of malignancy in the other organ systems. Twenty healthy participants were involved in our study. Written informed consent was obtained from each participant under a protocol approved by the Ethics Committee of the Eastern Hepatobiliary Surgery Hospital of the Second Military Medical University. Following the informed consent, sera from all individuals was collected for the evaluation of immunoglobulin.

**Table 1:** Clinical and biochemical characteristics of patients with chronic liver diseases.

Characteristics	Normal (n = 20)	Chronic liver disease	
		**Cirrhosis (n = 26)**	**HCC (n = 38)**
**Age (years, mean±SD)**	48.27±10.26	51.69±9.26	50±13.46
**Gender (male: female)**	15:5	18:8	29:9
**AFP (ng/ml)**	-	22.04±34.03	496.85±454.11
**ALT (IU/L)**	-	31.35±17.63	47.62±29.52
**HBV antigens (n; positive:negative)**	-	26:0	22:16
**Tumor size (cm)**	-	-	8.36±4.52
**Tumor numbers**	-	-	1.45±0.69
**Total globulins (g/l)**	23.89±2.89	38.28±4.37*	35.67±5.12*
**IgG (g/l)**	14.34±2.14	28.28±5.92*	22.85±6.73**
**IgM (g/l)**	1.48±0.72	1.46±0.74*	2.47±0.58*
**IgE (g/l)**	0.22±0.08	0.72±0.28**	0.61±0.31*

Mean ± SD, Student’s t test

*P<0.05

**P<0.01, comparison between patients with normal.

### Animal experiments

Eight-week-old male BALB/c mice weighing 28–30 g and eight-week-old male Sprague-Dawley (SD) rats with body weighs ranging from 230 to 250 g were obtained from the Shanghai Experimental Center, Chinese Academy of Science, and were maintained under specific pathogen-free conditions. Animals were housed at 22°C on a 12-h dark-light cycle with ad libitum access to food and water. Each experimental group contained a minimum of seven rats. A cirrhosis model was induced by intraperitoneal administrations of CCl4 (0.4 g/kg of body weight) dissolved in mineral oil three times per week for 8 weeks, or a 4-week bile duct ligation (BDL). The hepatocarcinogenesis model was induced by intraperitioneal injections of diethylnitrosamine (DEN; Sigma-Aldrich, St. Louis, MO) at 70 mg/kg once a week for 10 weeks in rats and at 25 mg/kg once in BALB/c mice when they were 15 days old; animals were then fed normally for 10–12 months. Additionally, an end-to-side portacaval shunt was performed on normal rats to mimic liver dysfunction. For gut sterilization, seven BALB/c mice were administered ampicillin (1 g/l; Sigma), neomycin sulfate (1 g/l; Sigma), metronidazole (1 g/l; Sigma) and vancomycin (500 mg/l; Abbott Labs) in their drinking water for 4 weeks. For the elevated plasma lipopolysaccharide (LPS) level, seven BALB/c mice received LPS (Sigma-Aldrich, St. Louis, MO; 1 mg/kg) by intraperitioneal injections for 24 hours. All rats and mice were anesthetized with 10% or 4% chloral hydrate, and blood samples were collected. The animals were euthanized at an indicated time. All experimental procedures involving animals were performed in accordance with the Institutional Animal Welfare Guidelines of the Eastern Hepatobiliary Surgery Hospital of the Second Military Medical University with protocol number: 13071002106.

### Immunization of animals

Portal venous blood and inferior vena cava blood were separately collected from all experimental rats and mice, and the sera were obtained by centrifuging the samples at 4000 rpm for 10 minutes. Then, 50 μl of sera were diluted in 450 μl of ddH_2_O. Healthy SD rats were immunized with 50 μl of the dilute serum and 450 μl 4% Al(OH)_3_ 5 times at two-day intervals, and mice were immunized with 25 μμl of the serum and 225 μl 4% Al(OH) _3_ 5 times at two-day intervals.

### Serum immunoglobulin ELISA assay

All sera samples from individuals, rats and mice were obtained from portal vena blood and inferior vena blood, and total immunoglobulins, IgM, IgG, IgE levels were determined using quantitative enzyme-linked immunosorbent assay (ELISA) (Immunology Consultants Laboratory, Inc. USA) according to the manufacturer’s instructions.

### BrdU incorporation

For the analysis of plasma cell proliferation, the mice were in vivo labeled by administering four intraperitoneal injections of bromodeoxyuridine (BrdU; Sigma, 1 mg/mouse/injection) spaced 4 hours apart, and the mice were euthanized 24 hours after the last injection. At that time, the spleen was ground and treated with Mouse 1X Lymphocyte Separation Medium to separate the lymphocyte cells. The lymphocyte cells were stained with PE-conjugated anti-CD138 and FITC-conjugated anti-BrdU for flow cytometry (FCM) analysis using the FITC BrdU Flow Kit (BD Pharmingen) according to the manufacturer’s instructions. The percentage of BrdU+ cells in the CD138 population was assessed.

### Limulus Amoebocyte Lysate Assay

Plasma lipopolysaccharide (LPS) concentrations were measured with a commercial kit (GenScript) according to the manufacturer’s instructions. Briefly, samples were diluted 1/10–1/500 with endotoxin-free water, adjusted to the recommended pH, and heated for 10 min at 70°C to minimize inhibition or enhancement by contaminating proteins. Limulus amebocyte lysate assay (LAL) reagents were added to the sample (in duplicate) and incubated at 37°C for 45 min, and the absorbance was read at 545 nm. All samples were validated for the recovery and internal coefficient variation using known amounts of LPS.

### Statistical analysis

All experiments were performed at least three times. Statistical analyses were performed using GraphPad Prism 5 (GraphPad Software, Inc., San Diego, CA, USA). Differences between the two groups were compared using the unpaired Student’s t test. *P* values less than 0.05 were considered to be statistically significant.

## Results

### Increased levels of serum immunoglobulin in patients with chronic liver diseases

**Fig 1:**
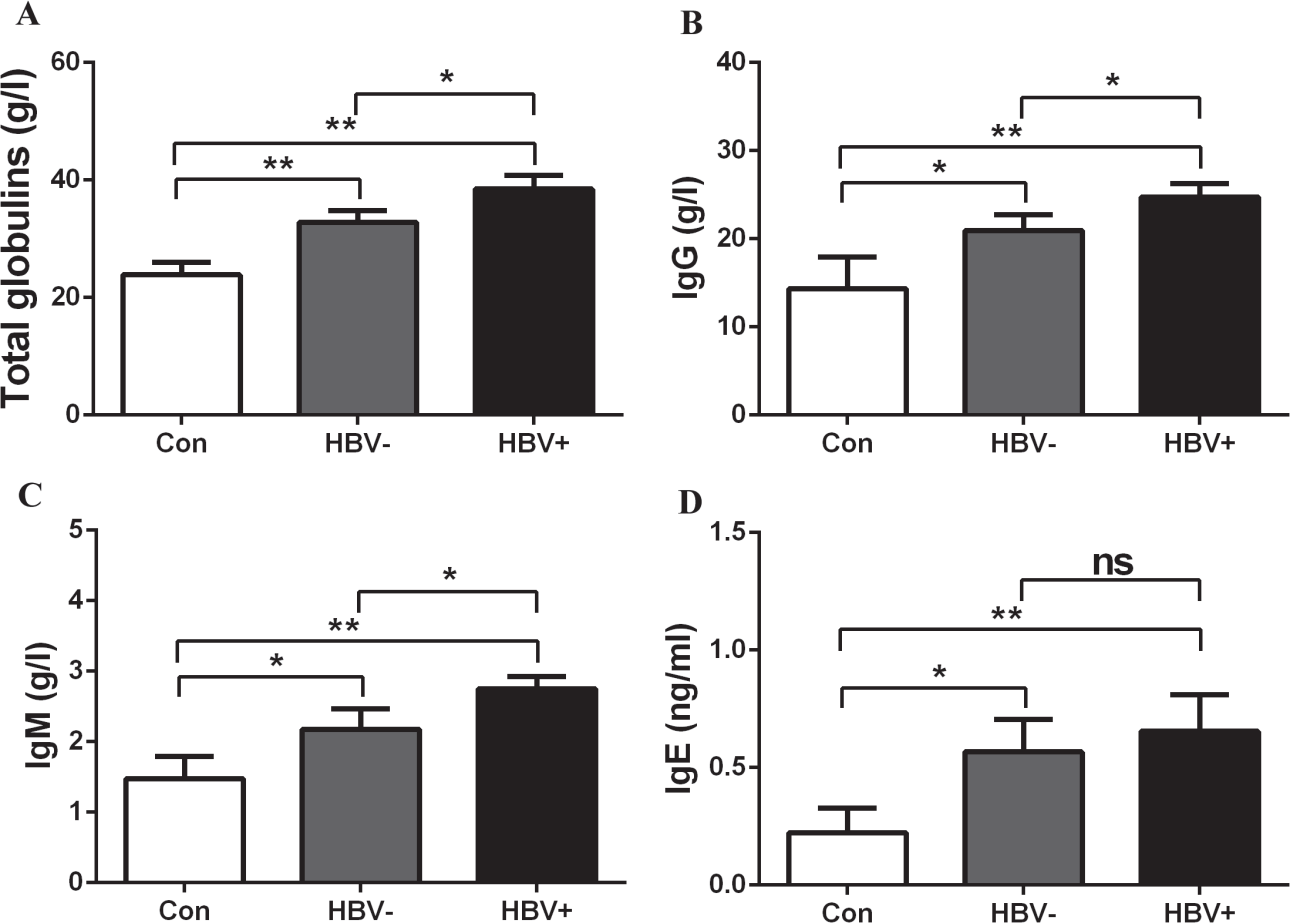
Increased levels of immunoglobulin were present in HCC patients with or without chronic hepatitis B virus infection. The levels of total immunoglobulin, IgG, IgM, IgE were evaluated in healthy controls, HBV- (HBV-negative) and HBV+ (HBV-positive) patients. *p< 0.05, **p<0.01, ns, no significant.

Serum total immunoglobulins and subtype IgG, IgM and IgE concentrations in the patients studied are presented in Table 1. As expected, serum total immunoglobulins, IgG, IgM and IgE levels were increased significantly in patients with cirrhosis and HCC compared to healthy donors (Table 1). Additionally, elevated levels of serum immunoglobulin were also detected in HBV-positive patients compared with HBV-negative patients. We found that HBV-negative patients with chronic liver diseases also showed high levels of immunoglobulin compared with healthy controls (Fig 1A–1D). The results indicate that chronic HBV infection is not the only cause of hyperimmunoglobulinemia.

### The injured liver increases immunogenesis by activating plasma cell proliferation

**Fig 2:**
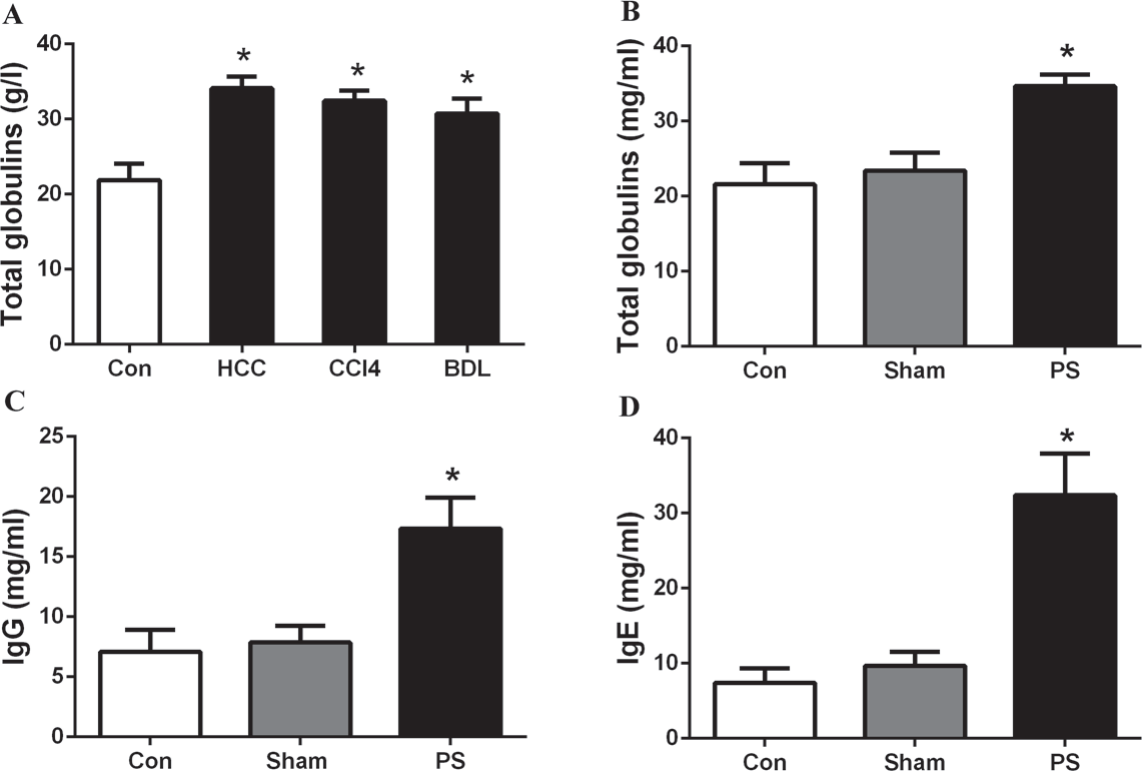
High levels of immunoglobulin in cirrhosis and HCC rats were in accordance with the clinical diagnosis of patients. (A) Total immunoglobulin was evaluated in control rats, DEN-challenged rats, and rats treated with CCl4 and bile duct ligation; (B, C, D) Total immunoglobulin, IgG and IgE were evaluated in control rats and those undergoing a sham operation or end-to-side portacaval shunt. PS, portacaval shunt. BDL, bile duct ligation. *p< 0.05.

**Fig 3:**
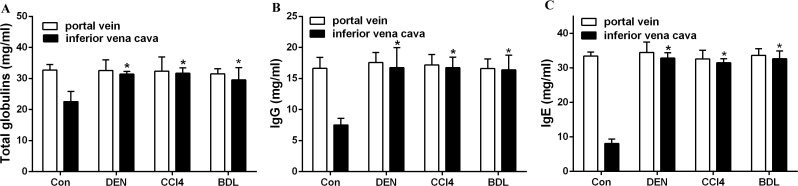
Inferior vena cava serum from rats with injured livers increased the production of immunoglobulin. Total immunoglobulin, IgG and IgE were evaluated in healthy rats that were immunized with serum from the portal vein and inferior vena cava from normal rats and rats treated with DEN, CCl4 and BDL, respectively. *p< 0.05.

**Fig 4:**
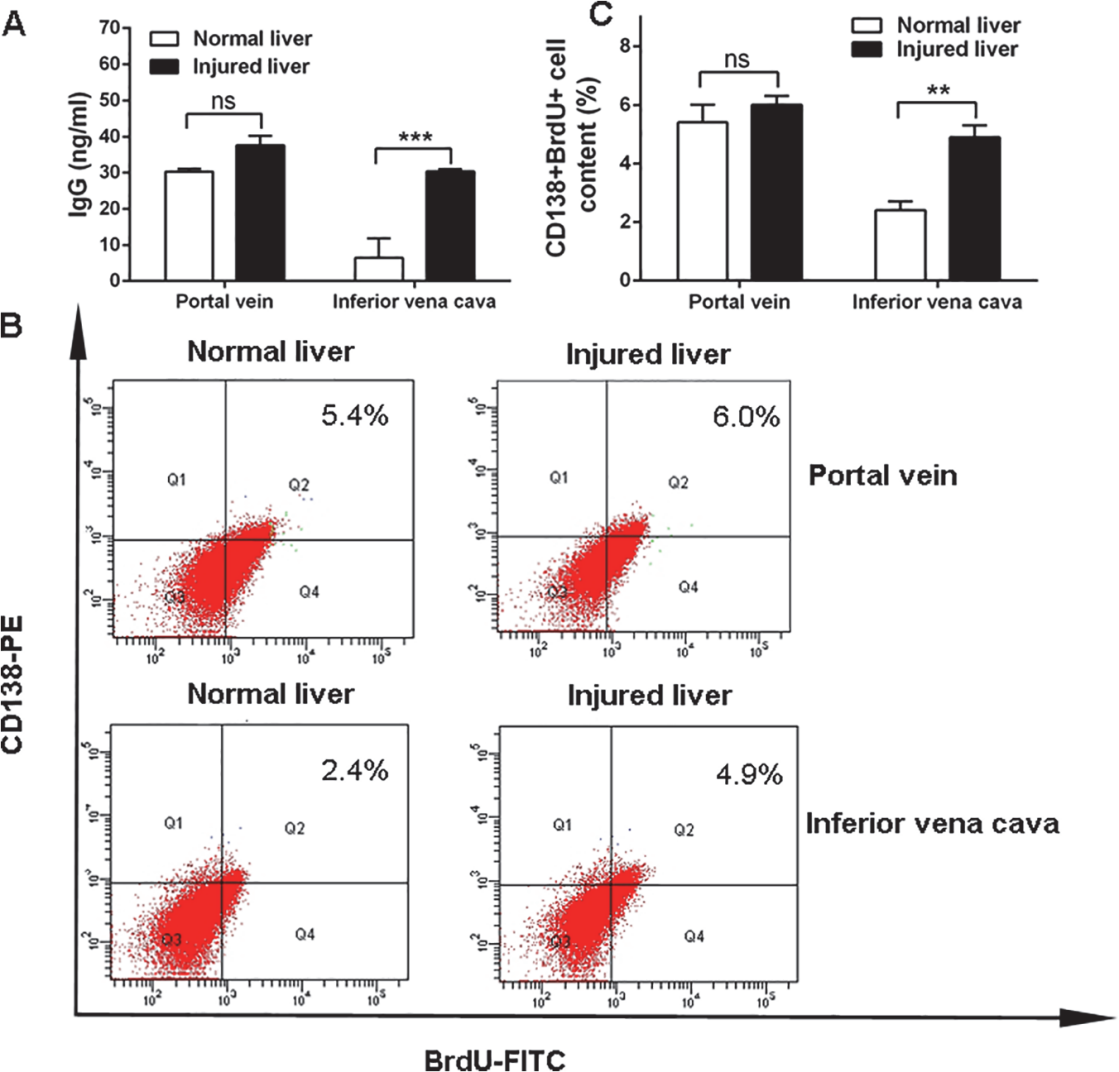
Inferior vena cava serum from mice with injured livers increased immunoglobulin synthesis by activating the plasma cell proliferation. (A) IgG was measured in healthy mice that were immunized with serum from the portal vein and inferior vena cava from mice with normal and injured livers (HCC mice), respectively. IgG was higher among mice immunized with serum from the inferior vena cava from HCC mice compared with normal mice. There were no significantly differences in IgG levels among mice treated with portal vein serum from HCC mice compared to mice treated with serum from normal mice. (B and C) The CD138+/BrdU+ cell population was analyzed by FACS analysis, and mice immunized with serum from the inferior vena cava of mice with injured livers showed a larger number of CD138+/BrdU+ cells (4.9%) than mice immunized with serum from the inferior vena cava of mice with normal livers (2.4%). No significant differences were shown in mice treated with portal vein serum from mice with normal liver and those with injured livers, three FACS samples were analyzed. ns, not significant, **p<0.01, ***p<0.001.

Increased levels of total immunoglobulin, IgG and IgE were shown in rats with cirrhosis and HCC, as well as in rats with an end-to-side portacaval shunt (Fig 2A–2D). The results are consistent with those from patients with liver disease. Normal rats demonstrated high antibody production after receiving serum from the portal venous blood or inferior vena cava blood of normal rats; elevated immunoglobulin levels were present in rats when they were injected with serum from the inferior vena cava blood of rats with liver disease (Fig 3A–3C). These phenomena were also demonstrated in mice, as higher levels of immunoglobulin were detected in mice challenged with serum from the inferior vena cava blood of HCC mice than those challenged with serum from the controls (*p*<0.001; Fig 4A); additionally, mice challenged with portal vein serum, which had not been processed by the liver, also developed hyperimmunoglobulinemia. The results indicate that the healthy liver may play a key role in the body’s immune response. Additionally, these immunized mice were also administered BrdU by intraperitoneal injection to mark the plasma cell “birthday”, and surface antigen CD138 was stained for the analysis of subsets of the plasma cell lineage cells. FCM analysis was then performed. We found a significant difference in plasma cell proliferation between mice that were immunized with inferior vena cava serum from mice with injured livers (4.9%) and mice with normal livers (2.4%) (*p*<0.01; Fig 4B and 4C). These results show that the diseased liver increases immunogenesis, which results in high immunoglobulin production by activating plasma cells proliferation.

### High plasma LPS levels were present because the injured liver cannot process LPS from the gut

**Fig 5:**
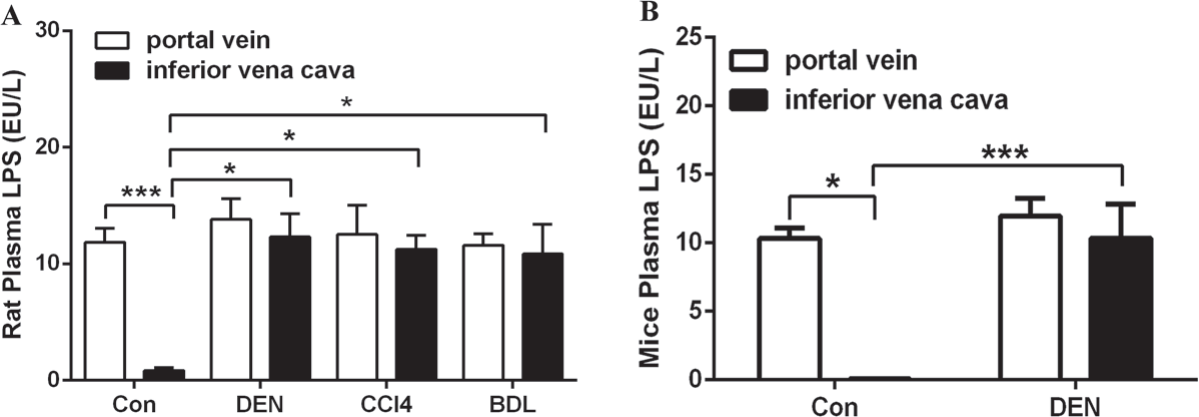
Plasma LPS levels in different rat and mouse models. Plasma LPS was determined in portal venous blood and inferior vena cava blood from normal rats and rats treated with DEN, CCl4 and bile duct ligation, as well as normal mice and DEN-treated mice. *p<0.05, ***p<0.001.

Plasma LPS level was also determined in portal venous blood and inferior vena cava blood from normal rats and those with cirrhosis and HCC, as well as from normal and HCC BALB/c mice. In normal rats, serum LPS levels were higher in portal vein blood than in inferior vena blood (*p*<0.001; Fig 5A), which was also shown in healthy mice (*p*<0.05; Fig 5B). A significant increase in LPS levels in the inferior vena cava blood of rats treated with diethylnitrosamine (DEN), CCl4 and BDL was detected (*p*<0.05; Fig 5A). LPS levels in inferior vena cava blood from DEN-treated mice were higher than in controls (*p*<0.001; Fig 5B). These results indicate that the diseased liver failed to process LPS, thus leading to elevated levels of plasma LPS.

### The diseased liver cannot dispose of antigens or endotoxins from the gut, which activates the proliferation of plasma cells

**Fig 6:**
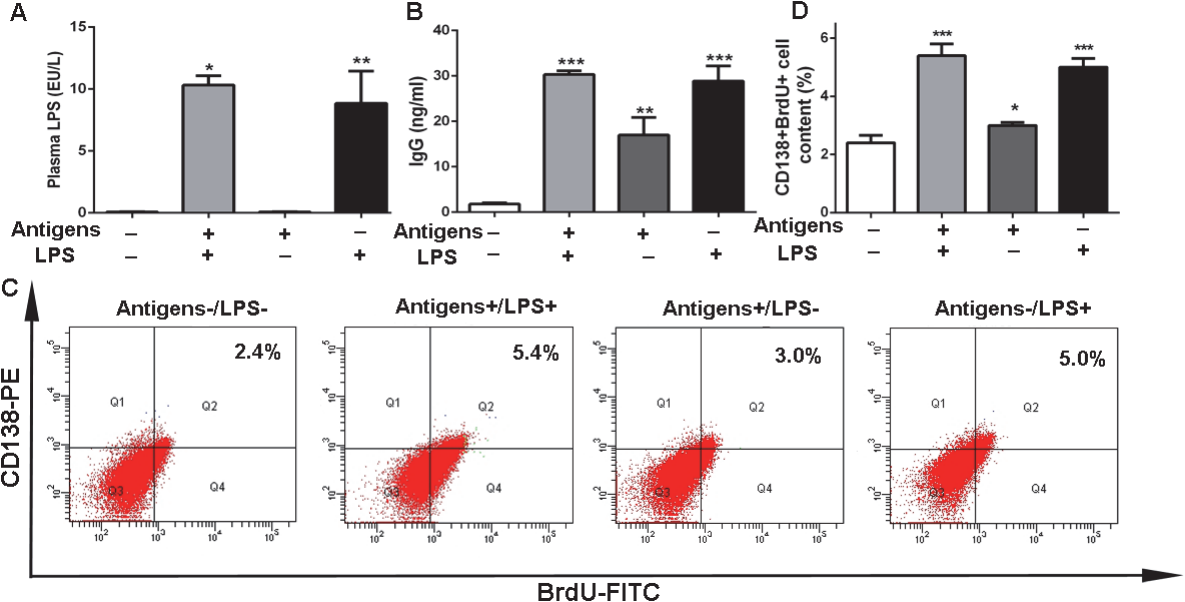
The diseased liver cannot dispose of antigens and endotoxins from the gut and activates the proliferation of plasma cells. (A) Plasma LPS level was measured in serum with different antigen and LPS levels (antigen-/LPS-, antigen+/LPS+, antigen+/LPS-, and antigen-/LPS+ serum). ++++(B) IgG was tested in mice immunized with antigen-/LPS-, antigen+/LPS+, antigen+/LPS-, and antigen-/LPS+ serum. (C and D) FACS analysis shows the proliferation of plasma cells (CD138+BrdU+) in antigen-/LPS-, antigen+/LPS+, antigen+/LPS-, and antigen-/LPS+ serum-challenged BALB/c mice, respectively, three FACS samples were analyzed. Antigen-/LPS-: serum from the inferior vena cava of healthy mice. Antigen+/LPS+: portal venous serum of healthy mice. Antigen+/LPS-: serum was separated from the portal venous blood of mice treated with antibiotics. Antigen-/LPS+: serum was collected from inferior vena cava blood of LPS-challenged mice. *p<0.05, **p<0.01, ***p<0.001.

To determine whether the elevated levels of immunoglobulin were associated with both the antigens and endotoxins derived from the gut, serum from mice treated with different regiments were collected. Serum from the inferior vena cava of healthy mice was used as the control (both antigens and endotoxins were negative, antigen-/LPS-). Portal venous serum of healthy mice was positive for both antigens and endotoxins (antigen+/LPS+). Portal venous serum of mice treated with antibiotics was antigen-positive and LPS-negative (antigen+/LPS-). Serum from the inferior vena cava was collected from LPS-challenged mice and was antigen-negative and LPS-positive (antigen-/LPS+) (Fig 6A). Healthy mice were immunized with the various serums, and IgG was examined following immunization. We found that antigen+/LPS+ serum was associated with significantly greater immunoglobulin levels than antigen-/LPS- serum (*p*<0.001; Fig 6B), and both antigens and LPS can induce the production of antibodies (Fig 6B). We then determined the fraction of cells in the S phage of the cell cycle in the spleen and the percentage of BrdU-labeled cells in this fraction. As shown in Fig 6C, a greater number of plasma cells (5.4%) became BrdU positive (BrdU+) in antigen+/LPS+ serum-treated mice, and 3.0% and 5.0% CD138+BrdU+ cells were shown in mice immunized with antigen+/LPS- and antigen-/LPS+ serum, respectively; all numbers were greater than in control (antigen-/LPS-treated, 2.4%, Fig 6C and 6D). These results reveal that both antigens and endotoxins have the ability to increase the production of serum immunoglobulin by activating the proliferation of plasma cells.

## Discussion

In the present study, we demonstrated that hyperimmunoglobulinemia in chronic liver disease is mainly associated with the diseased liver, which was confirmed by the high levels of immunoglobulin in the rats with an end-to-side portacaval shunt. The presence of elevated serum immunoglobulin levels was associated with a significant increase in plasma cell activation, which was confirmed by the significant increase of plasma cell proliferation in response to immunization with serum containing higher levels of antigens and endotoxins, which was obtained from the inferior vena cava blood of HCC mice. Thus, it appears that due to the collateral circulation secondary to portal hypertension, gut antigens and endotoxins bypass the liver and reach the antibody-producing cells.

The liver is an organ with predominant innate immunity and acts as an organ barrier or a filter between the digestive tract and the rest of the body. Innate immunity is also involved in the pathogenesis of liver cirrhosis [1]. The exact mechanism underlying the high levels of antibody-formation is not fully understood. Previous studies show that hyperimmunoglobulinemia in the systemic circulation is most closely related with chronic HBV infection [16–18]. In our study, we found that HBV-negative patients with chronic liver diseases also showed high levels of serum immunoglobulin, which means that the presence of elevated levels of immunoglobulin is associated with factors or mechanisms other than HBV infection. Triger and Wright indicated that the injured liver may lose its filtering capacity and allow many gut antigens and endotoxins to access the immune system, which would result in increased synthesis of immunoglobulins [20]. This was shown in our study by the elevated serum immunoglobulins in the rats, which was caused by an end-to-side portacaval shunt. This systemic immunization with enteric antigens and endotoxins may represent a “filtering” defect in the Kupffer cells or may simply be the shunting of antigens from the portal circulation into the systemic circulation.

Because the diseased liver is unable to handle these gut antigens and endotoxins, extrahepatic antibody-producing sites are activated. Earlier studies by Kent *et al* and Katz *et al* provide evidence for increased synthesis of serum immunoglobulins in the lymph nodes and spleen in both experimentally-acquired and naturally-acquired liver disease [21, 22]. In our study, the differences in the number of plasma cells in the spleens of mice immunized with serums with differing antigens and LPS status indicate that elevated immunoglobulin levels are reflected in an increase of the circulating plasma cell population. Structural changes in the intestinal mucosa occur in patients with chronic liver disease that allow for the translocation of bacteria and bacterial products, such as LPS, to the circulation [23]. The endotoxin moiety of LPS has been shown to be a B-cell mitogen, which shows effects of an anamnestic process [15, 24]. Toll-like receptors recognize bacterial products and evoke intense inflammatory reactions that might contribute to the pathogenesis of the damaged liver [25]. A potential mechanism involving TLR9 has been shown to cause increased IgA levels in patients with alcoholic liver disease [26], and TLR-7-mediated plasma cell activation has also been reported to induce Ig synthesis [27–29]. Therefore, it is possible that stimulation of plasma cells by Toll-like receptors in response to changes in the bacterial microflora and bacterial translocation is responsible for the high immunoglobulin levels seen in acute and chronic liver disease. We suggest that the presence of LPS and antigens activates Toll-like receptors, which then stimulate the memory cells of the B-lymphocyte group, and immunoglobulins are produced. Therefore, liver dysfunction causes activation and proliferation of circulating plasma cells, and the liver is unable to effectively dispose of the gut antigens and endotoxins, which results in markedly elevated levels of immunoglobulin.

## Conclusion

Elevated levels of serum immunoglobulin were present in patients with cirrhosis and HCC and were higher than the levels in healthy controls; this was also detected in the rat models of cirrhosis and HCC. Increased levels of immunoglobulin and proliferation of plasma cells were shown in mice treated with serum with high levels of antigens and endotoxins. We demonstrated that the clinical feature of increased serum immunoglobulin levels in patients with chronic liver disease was associated with liver dysfunction and the activation and proliferation of plasma cells.
